# Correlation between tumor engraftment in patient-derived xenograft models and clinical outcomes in colorectal cancer patients

**DOI:** 10.18632/oncotarget.3863

**Published:** 2015-04-18

**Authors:** Bo Young Oh, Woo Yong Lee, Sungwon Jung, Hye Kyung Hong, Do-Hyun Nam, Yoon Ah Park, Jung Wook Huh, Seong Hyeon Yun, Hee Cheol Kim, Ho-Kyung Chun, Yong Beom Cho

**Affiliations:** ^1^ Department of Surgery, Samsung Medical Center, Sungkyunkwan University School of Medicine, Seoul, Korea; ^2^ Department of Health Sciences and Technology, SAIHST, Sungkyunkwan University, Seoul, Korea; ^3^ Department of Genome Medicine and Science, Graduate School of Medicine, Gachon University, Incheon, Korea; ^4^ Department of Neurosurgery, Samsung Medical Center, Sungkyunkwan University School of Medicine, Seoul, Korea; ^5^ Department of Surgery, Kangbuk Samsung Hospital, Sungkyunkwan University School of Medicine, Seoul, Korea; ^6^ Department of Medical Device Management and Research, SAIHST, Sungkyunkwan University, Seoul, Korea

**Keywords:** colorectal cancer, survival, xenograft, genomic profile, drug response

## Abstract

Despite numerous studies involving patient-derived xenograft (PDX) models, few studies have investigated the relationship between the ability of the tumor to engraft (tumorigenicity) and the clinical features of colorectal cancer (CRC). The aim of this study was to determine whether tumorigenicity correlates with clinical outcomes of CRC patients. We included 241 CRC patients who underwent radical surgery from 2010 to 2013. PDX models were established by implanting tumor fragments obtained from these patients into the subcutaneous layer of immunodeficient mice. Xenografts were successfully established from 62.2%. Successful engraftment was associated with advanced stage (*p* < 0.001) and moderate/poor differentiation (*p* = 0.029). Three-year disease-free survival (DFS) rates were lower for patients with tumorigenicity (*p* = 0.011). In stage III patients, tumorigenicity was an independent predictor of poor DFS (*p* = 0.034). In addition, mutation of TP53 was most frequently detected in stage III patients with tumorigenicity. Two models of stage IV disease without KRAS mutations showed high sensitivity to EGFR-targeted agents, while none of the models with KRAS mutations showed high sensitivity. In conclusion, PDX models may provide an effective preclinical tool for predicting cancer progression and could be used to further genomic and pharmacologic research on personalized treatments.

## INTRODUCTION

Colorectal cancer is the third most common malignancy and a leading cause of cancer-related deaths worldwide [1-4]. Early diagnosis and treatment have improved survival of colorectal cancer, but the mortality rate is still the fourth highest in males and third highest in females [4, 5]. This high mortality rate is due to recurrence and metastasis, which occur in approximately 50% of patients during the course of disease [6, 7]. Surgical resection combined with systemic chemotherapy has improved survival rates in colorectal cancer, but treatment outcomes in patients whose disease has progressed remain unsatisfactory. Newer targeted agents such as cetuximab and panitumumab are widely used to treat metastatic colorectal cancer [8, 9]. However, some patients do not respond to these targeted therapies [10], indicating the need to develop personalized treatments for these patients.

Numerous molecular investigations have been carried out to develop personalized treatments [11], requiring models that accurately represent the biologic characteristics of the individual patient. Such preclinical studies have used cancer cell lines or patient-derived xenograft (PDX) models [10, 12]. Although cell lines are practical and easy to manipulate, they generally show poorly differentiated histology and lack similarity to the original tumor [13, 14], whereas PDX models better reflect characteristics of the original tumor, including tumor heterogeneity [12, 13]. As a result, PDX models have been widely used to develop treatment strategies for patients with refractory cancer. However, not all tumors specimens from cancer patients engraft successfully in animal models, and this difference may be associated with the progressiveness of the original tumor. Despite the increased use of PDX models, few studies have reported the relationship between engraftment of tumor specimens and clinical features of patients with colorectal cancer. This relationship may be useful in the interpretation of results in preclinical studies using PDX models. Therefore, in this study we evaluated the relationship between tumor engraftment in PDX models and clinical outcomes in patients with colorectal cancer.

## RESULTS

### Patient characteristics and tumorigenicity

Of the 241 patients with colorectal cancer, 135 were male and 106 were female. Median age was 59.9 years (range, 24–89). Patients were classified according to cancer stage: stage I (*n* = 15), stage II (*n* = 72), stage III (*n* = 84), and stage IV (*n* = 70). In patients with stage IV cancer, sites of metastasis included the liver (*n* = 61), distant lymph nodes (*n* = 4), lung (*n* = 3), ovary (*n* = 1), and peritoneum (*n* = 1).

Of the 241 tumor specimens, 150 (62.2%) successfully engrafted, reaching a size of 1,000 mm^3^ in 90 ± 20 days. The remaining 91 tumor specimens (37.8%) failed to engraft. Tumorigenicity according to patient characteristics is shown in Table 1. Tumor take rates were significantly higher for more advanced stage primary tumors (*p* < 0.001), with xenografts established from four of 15 (26.7%) stage I tumors, 41 of 72 (56.9%) stage II tumors, 50 of 84 (59.5%) stage III tumors, and 55 of 70 (78.6%) stage IV tumors. Tumor take rates were significantly higher for moderately differentiated (66.5%) and poorly differentiated (66.7%) tumors compared with well-differentiated tumors (46.7%, *p* = 0.029).

**Table 1:** Patient characteristics and tumorigenicity of primary tumors in PDX models

	Patients (n=241)	Tumorigenicity		
		Yes (n=150)	No (n=91)	p-value
Age, n (%)				0.432
< 60 years	114 (47.3%)	68 (59.6%)	46 (40.4%)	
≥ 60 years	127 (52.7%)	82 (64.6%)	45 (35.4%)	
Gender, n (%)				0.281
Male	135 (56.0%)	80 (59.3%)	55 (40.7%)	
Female	106 (44.0%)	70 (66.0%)	36 (34.0%)	
Preoperative CEA level				0.274
< 5 ng/ml	151 (62.7%)	90 (59.6%)	61 (40.4%)	
≥ 5 ng/ml	90 (37.3%)	60 (66.7%)	30 (33.3%)	
Primary tumor location, n (%)				0.744
Right colon	63 (26.1%)	38 (60.3%)	25 (39.7%)	
Left colon	121 (50.2%)	76 (62.8%)	45 (37.2%)	
Rectum	57 (23.7%)	36 (63.2%)	21 (36.8%)	
Tumor stage, n (%)				< 0.001
I	15 (6.2%)	4 (26.7%)	11 (73.3%)	
II	72 (29.9%)	41 (56.9%)	31 (43.1%)	
III	84 (34.9%)	50 (59.5%)	34 (40.5%)	
IV	70 (29.0%)	55 (78.6%)	15 (21.4%)	
Cell type, n (%)WD	45 (18.7%)	21 (46.7%)	24 (53.3%)	
MD	170 (70.5%)	113 (66.5%)	57 (33.5%)	0.029
PD	12 (5.0%)	8 (66.7%)	4 (33.3%)	
Others	14 (5.8%)	8 (57.1%)	6 (42.9%)	
Vascular invasion, n (%)				0.124
Negative	180 (74.7%)	107 (59.4%)	73 (40.6%)	
Positive	61 (25.3%)	43 (70.5%)	18 (29.5%)	
Lymphatic invasion, n (%)				0.134
Negative	147 (61.0%)	86 (58.5%)	61 (41.5%)	
Positive	94 (39.0%)	64 (68.1%)	30 (31.9%)	
Perineural invasion, n (%)				0.729
Negative	170 (70.5%)	107 (62.9%)	63 (37.1%)	
Positive	71 (29.5%)	43 (60.6%)	28 (39.4%)	
MSI status, n (%)				0.116
MSS	219 (90.9%)	133 (60.7%)	86 (39.3%)	
MSI	19 (7.9%)	15 (78.9%)	4 (21.1%)	
Unknown	3 (1.2%)	2 (66.7%)	1 (33.3%)	

PDX, patient-derived xenograft; CEA, carcinoembryonic antigen; WD, well differentiated; MD, moderately differentiated; PD, poorly differentiated; Others, mucinous carcinoma and signet ring cell carcinoma; MSI, microsatellite instability; MSS, microsatellite stable.

Among the 70 patients with stage IV tumors, 50 PDX models were established using paired xenografts from primary and metastatic liver tumors. Tumorigenicity appeared to be higher for metastatic lesions than for primary tumors (84.0% *vs*. 78.6%), but this difference was not significant (*p* = 0.456).

### Clinical outcomes and tumorigenicity

**Figure 1:**
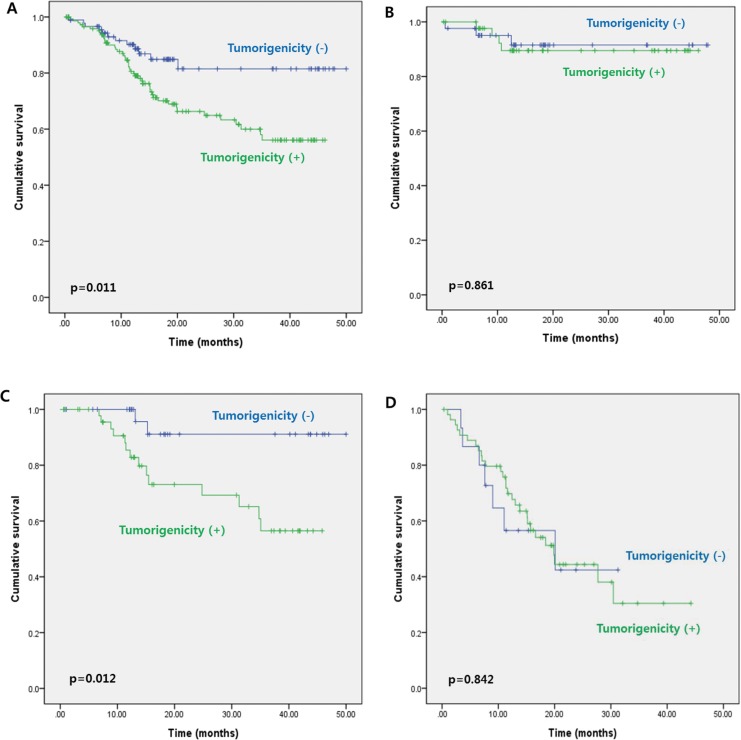
**Three-year disease-free survival according to tumorigenicity of the primary colorectal tumor for A.** all patients (stage I–IV cancer), **B.** patients with stage I–II cancer, **C.** patients with stage III cancer, and **D.** patients with stage IV cancer.

To better understand the relationship between PDX tumorigenicity and clinical outcomes, we analyzed the DFS of patients according to tumorigenicity. Median follow-up was 22.9 months (range, 0.2–51.3), and there were 58 recurrences and three deaths. The 3-year DFS rate of patients whose tumors successfully engrafted in the PDX model was significantly lower than that of patients whose tumors failed to engraft (56.1% *vs*. 81.5%, *p* = 0.011) (Figure 1A).

**Figure 2:**
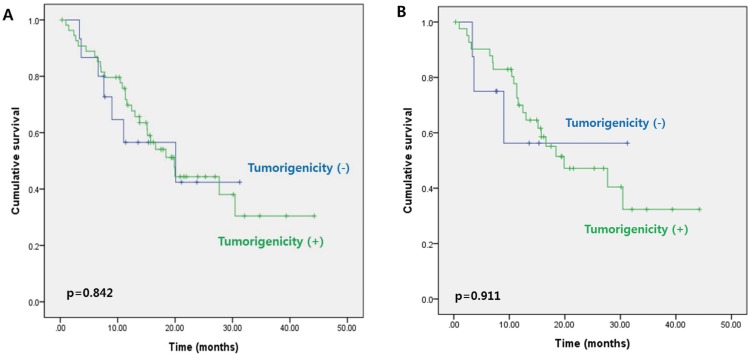
**Three-year disease-free survival in patients with stage IV colorectal cancer according to tumorigenicity of the A.** primary tumors and **B.** liver metastatic lesions.

Further analysis of 3-year DFS rates according to cancer stage showed no significant difference between patients with stage I–II cancer whose tumors failed to engraft and those whose tumors successfully engrafted (89.5% *vs*. 91.5%, respectively, *p* = 0.861) (Figure 1B). However, 3-year DFS was significantly lower for patients with stage III cancer whose tumors engrafted in the PDX model compared with those whose tumors failed to engraft (56.5% *vs*. 91.1%, *p* = 0.012) (Figure 1C). Results of multivariate analysis revealed that tumorigenicity in the PDX model (HR, 4.966; 95% CI, 1.126–21.905; *p* = 0.034) and old age (HR, 0.027; 95% CI, 1.178–14.600; *p* = 0.027) were independent predictors of DFS in patients with stage III cancer (Table 2). In patients with stage IV cancer, 3-year DFS appeared to be lower for patients whose tumors engrafted compared with those whose tumors failed to engraft (30.4% *vs*. 42.4%); however, this difference was not significant (*p* = 0.842) (Figure 2A). Similar results were obtained for the corresponding 50 liver metastatic lesions (32.3% *vs*. 56.3%, *p* = 0.911) (Figure 2B).

**Table 2:** Multivariate analysis of 3-year disease-free survival in patients with stage III colorectal cancer

Variable	p-value	HR (95% CI)
Tumorigenicity (+)	0.034	4.966 (1.126–21.905)
Age (≥ 60 years)	0.027	4.148 (1.178–14.600)

HR, hazard ratio; CI, confidence interval.

### Somatic DNA mutations of primary tumors with tumorigenicity

To investigate the mutational status of patients with tumorigenicity, we performed genomic profiling for successfully engrafted samples of primary tumor. We selected five tumor samples from patients with stage III disease, and analyzed the somatic DNA mutations of eight genes selected for their importance in colorectal cancer (Table 3). Nonsense and missense mutations were shown, where three of the five samples (60%) had transcription stop-gaining APC point mutations and four samples had TP53 point mutations (80%, one transcription stop-gaining mutation and four missense mutations). The mutation frequency of each gene was compared to that from 272 stage I-IV colon adenocarcinoma samples in The Cancer Genome Atlas (TCGA). The five samples from our study did not demonstrate KRAS mutation (0%), while 36% of TCGA samples had KRAS mutations. Four samples exhibited TP53 mutations (80%), while only 50% of TCGA samples had TP53 mutations. Even though the differences in mutation frequencies were not statistically significant due to the small sample size, further genomic profiling in future studies could elucidate these differences.

**Table 3:** Somatic DNA mutations of primary tumors from stage III patients

Gene	Patients					Mutation frequency (%)	TCGA mutation frequency (%)
	Pat-1	Pat-2	Pat-3	Pat-4	Pat-5		
APC	R536X	Q1285X	wt	R1096X	wt	60	67
FBXW7	wt	R387C	wt	wt	wt	20	12
TGFBR2	wt	wt	wt	wt	wt	0	4
PIK3CA	wt	wt	wt	wt	H1047R	20	22
KRAS	wt	wt	wt	wt	wt	0	36
BRAF	wt	wt	wt	wt	wt	0	13
ERBB2	wt	wt	wt	wt	wt	0	3
TP53	R81X	C143R	wt	R43H	R150W	80	50

Pat, patient; wt, wild type; TCGA, The Cancer Genome Atlas.

### Drug sensitivity to EGFR-targeted agents for PDX tumors

To investigate responsiveness to epidermal growth factor receptor (EGFR)-targeted agents according to mutation of EGFR-signaling genes, we performed genomic profiling for PDX tumors and drug sensitivity assay. Thirteen PDX models from nine patients with stage IV disease were selected for drug sensitivity testing. Somatic DNA mutations of selected genes, which either had known importance in colorectal cancer or belonged to the EGFR signaling pathway, were identified. Sensitivities to EGFR-targeted agents are presented for each model (Table 4). Nonsense and missense mutations are also presented, where two of 13 models showed high sensitivity to the EGFR-targeted agents, and both models belonged to the group without somatic KRAS mutations. None of the models with somatic KRAS mutations showed high sensitivity to the EGFR-targeted agents. Clearer correlation between the genomic profile of EGFR-signaling genes and responsiveness to EGFR targeted treatments can be shown with further enriched genomic profiles in the future.

**Table 4:** Somatic DNA mutations and drug sensitivity to EGFR targeted agents for PDX tumors

Patients	Tumor site	EGFR-signaling genes			Key genes of colorectal cancer						Drug sensitivity
		EGFR	KRAS	BRAF	APC	FBXW7	TGFBR2	PIK3CA	ERBB2	TP53	
Pat-6	Meta	wt	G13D	wt	R283X, E1286X	R689Q, R479Q	wt	wt	wt	wt	Low
Pat-7	Meta	wt	G12C	wt	R858X	wt	wt	wt	wt	wt	Low
Pat-8	Pri	wt	G12V	wt	K1543X, K1525X	wt	wt	wt	wt	G245S	Low
	Meta	wt	G12V	wt	K1543X, K1525X	wt	wt	wt	wt	G245S	Low
Pat-9	Meta	wt	G12D	wt	wt	wt	wt	wt	wt	wt	Low
Pat-10	Pri	wt	G12D	wt	E1286X	wt	wt	wt	wt	wt	Low
	Meta	wt	G12S	wt	E1286X	wt	wt	wt	wt	wt	Low
Pat-11	Pri	wt	wt	wt	R405X, Q789X, R1450X	wt	wt	wt	wt	R248Q	Low
	Meta	wt	wt	wt	R405X, Q789X, R1450X	wt	wt	wt	wt	R248Q	High
Pat-12	Pri	wt	wt	wt	R216X	wt	wt	wt	wt	wt	Low
Pat-13	Pri	wt	wt	wt	wt	wt	wt	wt	wt	wt	High
	Meta	wt	wt	wt	wt	wt	wt	wt	wt	wt	Low
Pat-14	Pri	wt	wt	wt	wt	wt	wt	wt	wt	wt	Low

Pat, patient; Pri, primary tumor; Meta, metastatic tumor; wt, wild type.

## DISCUSSION

In this study, we evaluated the relationship between successful tumor engraftment in PDX models and the clinical features of patients with colorectal cancer. Similar to the results of previous studies [10, 15], xenografts were successfully established with 62.2% of the primary tumors. Successful engraftment of the primary tumors was associated with advanced cancer stage and moderately/poorly differentiation. Primary tumor engraftment also appeared to be associated with higher preoperative carcinoembryonic antigen levels, vascular or lymphatic invasion, and microsatellite instability. For patients with stage IV cancer, the rate of engraftment was higher for metastatic lesions than for primary lesions. These results suggest that colorectal cancers with more aggressive features are better able to engraft than less aggressive cancers. In our study, 3-year DFS rates were lower for patients whose tumors successfully engrafted in the PDX model compared with patients whose tumors failed to engraft. However, when these patients were classified by cancer stage, stage III cancer showed a significant association between tumor engraftment and decreased DFS. In addition, tumorigenicity was found to be an independent predictor of DFS in stage III cancer. These findings suggest that patients with stage III cancer whose tumors successfully engraft in PDX models have an increased risk of relapse.

Few studies have evaluated the relationship between successful tumor engraftment in a PDX model and clinical outcomes in colorectal cancer [10, 12]. In a previous study to evaluate drug-response and tumor progression using 32 PDX models, tumor take rates were higher for poorly differentiated tumors and tumors from patients with lymph node invasion; however, survival did not differ among patient groups [12]. In another study, the authors compared tumor take rates of primary (*n* = 58) and metastatic (*n* = 27) lesions, reporting higher tumor take rates for metastatic lesions; however, this difference was not significant [10]. In our study, which analyzed a larger sample size than previous studies, we evaluated the relationships between tumor engraftment in the PDX model and patient characteristics and DFS. Similar studies have been carried out for non-small cell lung cancer, breast cancer, and uveal melanoma [11, 14, 16, 17]. John *et al*. reported that tumorigenicity correlated with the presence of KRAS mutations, poor differentiation, and larger tumor size in non-small cell lung cancer. In addition, tumorigenicity was an independent predictor of shorter DFS [14]. In contrast, Anderson *et al*. reported that tumorigenicity did not correlate with clinical outcomes in non-small cell lung cancer [16].

Cancer cell lines and PDX models have been widely used in the development of personalized cancer treatments. Xenografts derived from cell lines are reproducible, easy to manipulate, and well characterized; however, they do not exhibit tumor heterogeneity or the histopathologic and genetic characteristics of the tumor [8, 13, 14]. Numerous genomic mutations have been detected in patients with cancer, and high levels of oncogene mutation can accelerate the growth of tumors [18, 19]. In our study, we performed genomic profiling for primary tumor cells from patients whose tumors were successfully engrafted in the PDX models. We detected several mutations of colorectal cancer-related genes, and mutation of TP53 was the most frequently detected. According to several reports, PDX models better reflect the genetic diversity of the original tumor, and better predict clinical tumor response to new therapeutics [8, 12, 13, 20, 21]. Drug sensitivity assay with genomic profiling using PDX models can provide more information about primary tumors and new therapeutic strategies for patients, especially those with disease refractory to conventional treatments. In this study, we investigated somatic DNA mutations and accompanying sensitivity to EGFR-targeted agents in PDX models derived from patients with stage IV disease. Initial genomic profiling of PDX models and evaluation of EGFR-targeted treatments showed selective responsiveness depending on KRAS mutation. This is consistent with previous studies indicating that the mutation of EGFR-downstream KRAS can increase resistance to EGFR-targeted treatments [22-24]. However, responsiveness to EGFR-targeted agents can depend on several other factors not addressed in this study, which include DNA copy number changes, methylation status, and gene/protein expression levels. Available genomic profiles along with correlated drug response information significantly increases the applicability of PDX models, and further research is needed to complete the genomic and drug-response profiles of our PDX models.

PDX models are important for the development of novel treatments, especially for patients with refractory cancer, indicating the need for well-characterized xenograft models. In this study, our models stably established and we found that successful engraftment of patient-derived colorectal cancer cells correlates with more aggressive characteristics and worse outcomes. Therefore PDX models may provide an effective preclinical tool to evaluate cancer progression and treatment strategies. In addition, these models can be used for further genomic and pharmacologic studies to personalized treatments. Our results provide evidence that PDX models are applicable to colorectal cancer patients with a progressive disease course and high risk of relapse. We anticipate that personalized treatments using PDX models will improve survival rates for these patients.

In conclusion, our findings show that the successful engraftment of colorectal cancer tumors in PDX models is associated with more aggressive disease and worse clinical outcomes. Our PDX models maybe useful to predict disease progression in preclinical studies for personalized medicine and to improve clinical outcomes of patients. Further studies for genomic and pharmacologic information will provide novel treatments for patients with colorectal cancer.

## MATERIALS AND METHODS

### Patients

This study was approved by the Samsung Medical Center Institutional Review Board (no. 2010-04004). A total of 241 patients with colorectal cancer who underwent surgery from March 2010 to April 2013 at the Samsung Medical Center were included. All patients had histologically confirmed primary adenocarcinoma and underwent radical surgery for the primary tumor and synchronous metastatic lesions. Patients who underwent palliative operations and those with recurrent disease or synchronous malignancies were excluded.

### Tumor samples

Specimens from primary and metastatic tumors were obtained from patients who had provided written informed consent. Tumor tissues not required for clinical diagnosis were placed in Roswell Park Memorial Institute (RPMI) medium supplemented with 250 U/ml penicillin and 250 μg/ml streptomycin. Each tumor sample was cut into 5- to 10-mm^3^ pieces, some of which were snap frozen in liquid nitrogen and stored in a freezer at −80°C or in liquid nitrogen for molecular analysis. Two pieces were fixed in formalin solution and paraffin-embedded for histopathologic analysis, and two pieces were coated in high concentration Matrigel (BD Biosciences, Erembodegem, Belgium) and implanted in 6- to 8-week-old female Balb/c nude mice (Orient Bio, Seongnam, Korea). A similar process of sample preservation was carried out for tumor tissues collected from mice.

### Establishment of PDXs

Establishment of PDXs was performed as previously described [8]. Briefly, Matrigel-embedded tumor fragments (1–2 mm^3^) were implanted into subcutaneous pockets made in each side of the lower back. Tumors that reached a volume of 1,000 mm^3^ were considered tumorigenic. All animal experiments were carried out according to protocols approved by the appropriate institutional review boards of the Samsung Medical Center (K-B2-036) and conducted in accordance with the Institute for Laboratory Animal Research Guide for the Care and Use of Laboratory Animals.

### Genomic profiling of patients and PDX tumors

Liquid nitrogen-preserved samples from patients and PDXs were mechanically dissociated and genomic DNA was extracted using the QIAamp DNA mini kit (Qiagen, Valencia, CA). Agilent Sureselect Human All Exome Kit v4 was used to capture the exon region from DNA. To obtain DNA for patients' normal control blood cells, data from raw sequencing reads was produced by Illumina HiSeq^TM^2500. To obtain the DNA of tumor tissues from patients and PDXs, raw sequencing reads were produced by Illumina HiSeq^TM^2000. This resulted in mean coverage rates in the exome region of around 120x for the tumor tissues and around 80x for normal blood cells. Somatic mutations in tumor DNA were identified using the next generation sequencing (NGS) pipeline at Samsung Genome Institute, Seoul, Korea.

### Drug sensitivity assay of PDX tumors

Dissociated tumor cells from PDXs were grown in modified neurobasal A medium (Invitrogen, Carlsbad, CA) containing N2 supplement (Invitrogen), bFGF (20 ng/ml, Invitrogen), and EGF (50 ng/ml, Invitrogen). Tumor cells were seeded in 96-well plates at 1,000 cells per well, and treated with EGFR-targeted agents (Selleckchem, Houston, TX, USA) - AEE788, Afatinib, BMS-599626, Canertinib, CO-1686, Dacomitinib, Erlotinib, Gefitinib, Lapatinib, Neratinib - under seven-point serial dilution concentrations up to 20 μM (*n* = 3 for each condition). After three days of incubation at 37°C in a 5% CO_2_ humidified incubator, cell viability was analyzed using the metabolic conversion of a water-soluble tetrazolium salt, WST-1 (Roche, Indianapolis, IN). Plates were analyzed with a spectrophotometer at 450 nm, with a reference wavelength of 630 nm. For each drug-sample pair, the drug response curve was approximated using GraphPad Prism 6.0 (GraphPad Software, San Diego, CA, USA) based on the measured cell viabilities from varying drug concentrations.

### Clinicopathologic analysis

The tumor take rates of primary tumor specimens were analyzed according to patient characteristics. In addition, patients were grouped according to cancer stage, and survival rates of each group were analyzed according to tumorigenicity (i.e., tumor engraftment in the PDX model). In stage IV cases, survival rates were evaluated according to tumorigenicity of the primary tumors and corresponding liver metastatic lesions. The primary endpoint of this study was clinical outcome according to tumorigenicity. The secondary endpoint was response of PDX tumors to EGFR-targeted agents according to mutational status.

### Statistical analysis

Statistical analysis was performed using SPSS for Windows version 20.0 (IBM SPSS Statistics, IBM Corporation, Armonk, NY). Patient characteristics were compared using the chi-squared test or linear-by-linear association. Survival rates were analyzed using the Kaplan–Meier method and log-rank test. Multivariate analysis was performed using logistic regression to identify predictors of survival. Factors that were significant or near significant (*p* < 0.1) in univariate analysis were included in the multivariate model; *p* < 0.05 was considered significant.
